# The Impact of Selenium Exposure During Pregnancy on Risk for Miscarriage: A Systematic Review

**DOI:** 10.3390/ijms27020968

**Published:** 2026-01-18

**Authors:** Stavroula-Ioanna Kyriakou, Ermioni Tsarna, Nikolina Stachika, Christina Dalla, Anastasios Potiris, Sofoklis Stavros, Panagiotis Christopoulos

**Affiliations:** 1 Second Department of Obstetrics and Gynecology, “Aretaieion” University Hospital, Medical School, National and Kapodistrian University of Athens, 11528 Athens, Greece; stavrianna.kyr@gmail.com (S.-I.K.); ermioni.tsarna@gmail.com (E.T.); nnikolinast@gmail.com (N.S.); cdalla@med.uoa.gr (C.D.); 2 Third Department of Obstetrics and Gynecology, University General Hospital “ATTIKON”, Medical School, National and Kapodistrian University of Athens, 12462 Athens, Greece; apotiris@med.uoa.gr (A.P.); sfstavrou@med.uoa.gr (S.S.)

**Keywords:** selenium, pregnancy, miscarriage, abortion, pregnancy loss, oxidative stress, ferroptosis, immune system

## Abstract

Selenium (Se) is an antioxidant essential trace element influencing inflammatory and immune pathways. This systematic review aimed to evaluate the role of maternal Se status during pregnancy in miscarriage risk. A systematic search of PubMed and Embase up to July 2024 was conducted to identify relevant original research studies in English. Available evidence was qualitatively synthesized and predefined sources of bias were assessed. Of 2345 studies identified, 421 full texts were assessed and 14 were included, encompassing 2309 pregnancies. Despite notable methodological limitations across several studies, current evidence indicates that maternal blood Se concentrations are lower among women who experience miscarriage compared to those with uncomplicated pregnancies. Findings regarding placental Se levels were inconsistent, but important methodological issues were noted. Environmental Se exposure was investigated in a single low-powered study, which did not demonstrate a statistically significant association. Potential interactions between Se status, co-exposure to other environmental or lifestyle factors, and effect modification remain insufficiently explored. Adequate maternal Se status during early gestation may reduce miscarriage risk by mitigating oxidative stress and ferroptosis, supporting immune regulation, and modulating thyroid autoimmunity and function. However, causal inference cannot be established due to the absence of randomized interventional evidence.

## 1. Introduction

Selenium (Se) is an essential trace element that is omnipresent in nature. The nutritional status of Se in human organisms varies depending, among other things, on Se intake, gender, age, and bioavailability. Se adequacy has been implicated in the pathogenesis of cancer, immunity, brain development, and cardiovascular diseases [1]. Low Se levels are associated with increased risk of mortality, poor immune function, and cognitive decline [2]. On the other hand, excessive Se can cause selenosis, a condition that describes Se related toxicity [3].

Selenium as such is biologically inactive; its levels can be measured in several biological samples, including serum, whole blood plasma, red cells, placental samples, amniotic fluid, urine, and hair samples. In humans and other animals, Se occurs in organic compounds, such as selenomethionine and selenocysteine, which are the biologically functional forms of Se incorporated in proteins, referred to as selenoproteins [4]. In humans, selenoprotein P (SELENOP) is the primary selenoprotein in plasma and can be used as an index of sufficient Se intake [4]. It functions both as a Se transporter to peripheral tissues and as an antioxidant, protecting lipoproteins from oxidative damage [4]. Glutathione peroxidases, otherwise known as GPx enzymes, are also selenoproteins that neutralize peroxides, protecting cells from oxidative damage [4]. GPx activity is commonly assessed as a functional indicator of Se sufficiency; it rises in response to increasing Se concentrations but reaches a plateau once the enzyme system is saturated, rendering it insensitive to further increases in Se exposure [4]. GPx3 activity, which is mainly in plasma and thus reflects short-term Se status, typically reaches maximal levels at plasma Se concentrations between 70 and 90 µg/L [4,5]. GPx1 activity, which is mainly in red blood cells and platelets and reflects long-term Se status, plateaus at slightly higher Se levels (around 80 to 120 µg/L), although results vary across studies [4,5]. In contrast to GPx, plasma SELENOP concentration continues to rise until reaching ~110 µg/L Se in plasma, which is higher than the GPx plateau point [4]. Therefore, SELENOP may be a more sensitive marker for identifying suboptimal versus optimal Se status. GPx activity can also be measured in placental samples; it has been shown that enhanced placental GPx activity provides local protection against reactive oxygen species (ROS) activity and stimulates placental differentiation [6]. Among GPx enzymes, GPx4 is the only antioxidant enzyme in mammals that can inhibit the lipid peroxidation process and stabilize the cell membrane by converting the lipid peroxides into lipid alcohols, which are non-toxic [7]. In addition to its role in oxidative stress, GPx4 protects cells against ferroptosis, while its deficiency contributes to immune system dysregulation and increased inflammation [7]. GPx4 is located in the cytoplasm, the mitochondria, and the sperm nucleus in different isoforms [7].

Miscarriage refers to spontaneous pregnancy loss before 20 weeks of gestation. Overall, 10% to 20% of clinically confirmed pregnant women will miscarry [8]. When pregnancy loss happens during the first trimester, some organizations define it as an early miscarriage [9]. In particular, the American College of Obstetricians and Gynecologists (ACOG) defines miscarriage as “an empty gestational sac or a gestational sac containing an embryo or fetus without fetal heart activity within the first 12^6/7^ weeks of gestation”, while the European Society of Human Reproduction and Embryology (ESHRE) considers an early loss when occurring before 10 weeks of gestation [8,10]. Fetal chromosomal abnormalities are the most common cause of miscarriage and explain about 60% of the cases of pregnancy loss between 6 to 10 weeks of gestation [8,11,12]. Nonetheless, it appears that inflammatory and immunologic dysregulation also plays a key role in miscarriage pathogenesis due to their impact on trophoblastic invasion and placental development [13,14].

Considering the role of Se as an antioxidant and its interactions with both inflammation and immune function, a potential association between maternal Se status in early pregnancy and the risk of miscarriage can be postulated. The objective of this systematic review was to examine the relationship between Se status and/or Se supplementation in pregnant women and the incidence of miscarriage.

## 2. Materials and Methods

The Selenium Exposure during Pregnancy Project (SEduP) was designed to comprehensively evaluate existing evidence on the association between maternal Se status and pregnancy or neonatal outcomes. These include complications during gestation and neonatal events occurring within the first month of life. Maternal Se status was assessed in the literature either via supplementation studies or by quantifying Se or selenoprotein levels in biological specimens such as serum, plasma, whole blood, urine, amniotic fluid, and keratinous tissues (hair and nails). For inclusion, we considered only original research articles. Exclusion criteria encompassed case reports, narrative or systematic reviews, editorials, and letters to the editor that did not provide primary data. Additionally, studies involving animal models and publications not written in English were omitted from the qualitative synthesis.

A comprehensive search strategy was initially developed for PubMed and subsequently adapted for Embase using the “Polyglot” from the Systematic Review Accelerator (Table S1) [15,16]. The PubMed search incorporated MeSH terms and relevant keywords pertaining to pregnancy and Se, while employing a filter to safely exclude animal studies (Table 1). Search was performed in July 2024 and results were imported into the Rayyan platform [17], where duplicate entries were automatically flagged and manually verified before removal.

Following deduplication, two reviewers (S.K. and N.S.) independently screened all remaining records at the title and abstract level against the predefined inclusion and exclusion criteria. Studies deemed potentially eligible proceeded to full-text review by the same reviewers, again in a blinded and independent manner. Discrepancies during screening were resolved through consultation with a third reviewer (E.T.), as per study protocol. A structured labelling system was implemented to categorize the outcomes assessed in each included study. For this systematic review, we focused specifically on studies reporting on miscarriage risk among pregnant women, if their Se levels had been measured or they had received Se supplementation. Additionally, reference lists of included studies were hand-searched to identify relevant publications not captured by the databases search.

Data extraction was performed by one reviewer (S.K.), with a second reviewer (E.T.) independently verifying the accuracy of the extracted information. Where studies addressed multiple outcomes, cross-validation of Summary of Findings (SoF) tables for different outcomes was undertaken to ensure consistency. The SoF table for risk of miscarriage included details such as first author and publication year, geographic location of the study, study design, publication type (peer-reviewed article or conference abstract), sample size, period of data collection, intervention type and duration (for supplementation studies) or timing of Se levels assessment, details regarding past history of miscarriage, statistical methodologies employed, and the key findings (Table S2). The extracted information was then qualitatively synthesized to draw conclusions regarding the potential effect of Se on miscarriage risk. Results are presented grouped by the samples at which Se levels were measured (e.g., tap water, placenta, maternal blood).

With regard to sources of bias in individual studies and sources of heterogeneity in results between studies, we have pre-identified the following: differences in gestational age at sampling and maternal age, impact of co-exposure (e.g., other heavy metals) and lifestyle factors (e.g., smoking), different Se levels in study population, statistical power as captured in sample size. Furthermore, a group meeting was conducted after data collection and tabulation and the potential of additional sources of bias was explored by examining the variables that have been accounted for in each individual study. Reporting bias could not be assessed, since reporting on the association of Se status during pregnancy and miscarriage risk was among our inclusion criteria. Lastly, confidence in the body of evidence was assigned in a group meeting based on coherence across studies and consistency in the direction of results, identified methodological limitations including risk of bias in each individual study, and precision of the reported effect estimates. This systematic review follows PRISMA guidelines (Tables S3 and S4).

## 3. Results

**Figure 1:**
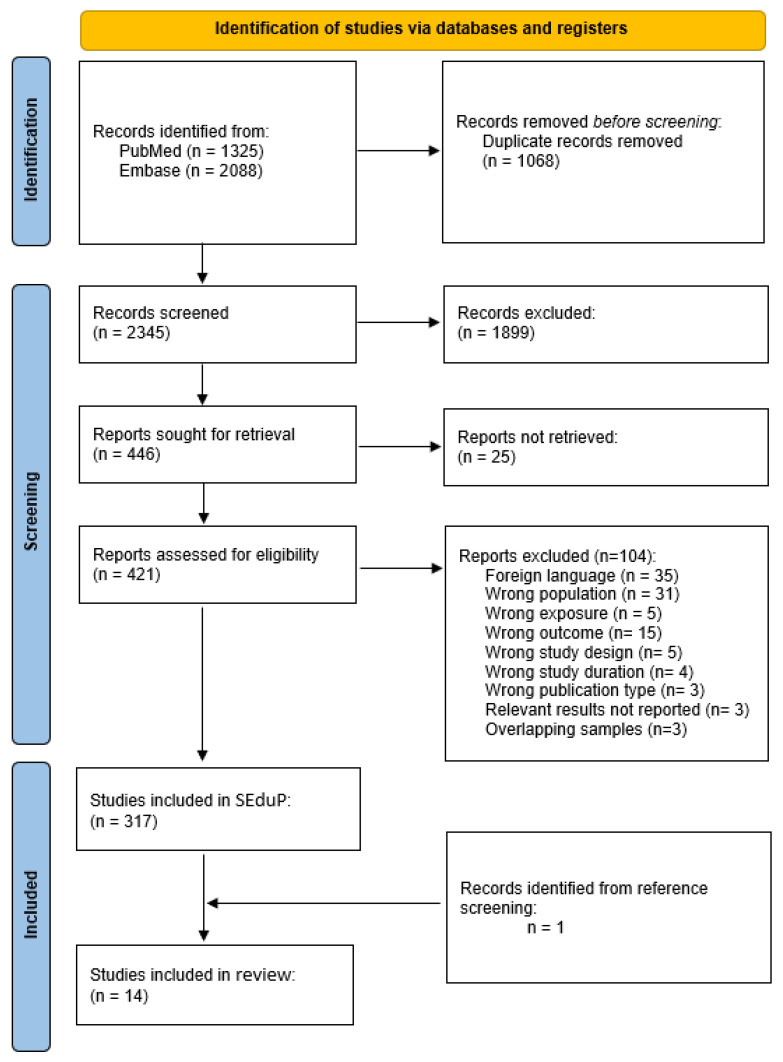
Flow Diagram of Studies Included in the Systematic Review.

The search strategy retrieved 1325 records from PubMed and 2088 from Embase. After removing 1068 duplicate records, 2345 titles and abstracts were screened for relevance. Of these, 1899 were excluded due to lack of relevance, leaving 446 articles for full-text review. Twenty-five articles could not be retrieved, and 104 were excluded following full-text assessment. Ultimately, 317 studies were retained in the SEduP database. For this systematic review, 14 studies met the inclusion criteria [18,19,20,21,22,23,24,25,26,27,28,29,30,31]; 13 were included in SEduP database and one was identified by hand-searching the reference lists. The study selection process is summarized in the PRISMA flow diagram (Figure 1).

Out of the 14 studies in this systematic review, one was a retrospective cohort from Italy that examined the impact of exposure to tap water with a high concentration of inorganic Se, including in total 1128 pregnant women, of whom 23 were regarded as exposed. The remaining 13 studies were all case-control in design and measured Se levels in different biological samples. In total, 1181 pregnant women were included in these studies, of whom 584 experienced a miscarriage, while 597 served as controls. Twelve studies measured the Se in maternal biological samples, mainly whole blood, serum, or plasma, while four studies examined Se levels in placental samples. With regard to geographical region, two studies were conducted in China, two in India, one in Indonesia, two in Saudi Arabia, and three in Turkey, while three studies were conducted in Europe (two in Poland and one in South Wales).

In the single study that did not measure Se levels in biological samples, higher incidence of miscarriage was observed in the exposed group [18]. However, the adjusted for maternal age odds ratio (OR) was not statistically significant (1.73, 95%CI 0.62–4.80). It should be noted that the number of exposed pregnancies was very small (*n* = 23) leading to low statistical power, and that the concentration of inorganic Se in tap water was well below the WHO limit of 40 μg/L both in the exposed (7–9 μg/L) and the unexposed (1 μg/L) pregnancies (Table 2) [32].

With regard to studies that examined the association of placental Se levels with miscarriage, results are conflicting, with two studies indicating higher Se levels in placentas of pregnant women that miscarried [20,26], while two studies reported lower levels (Table 2) [25,29]. However, significant methodological issues in all four studies indicate that confounding is likely present in their results, hindering their interpretation. Most importantly, placental samples from all pregnancies that served as controls were obtained at delivery; thus, gestational age differed between cases and controls. Therefore, the reported associations may reflect the difference in gestational age rather than a real difference between miscarriage cases and control pregnancies. Furthermore, in the study by Al-Sheikh et al., confounding due to maternal age may additionally have affected the reported results, since miscarriage cases were older than controls [29]. Lastly, Omeljaniuk et al. reported a positive correlation of BMI and Se levels in placenta, indicating the possibility of additional confounding by BMI [26]. To be noted, the two studies from Saudi Arabia that reported a protective effect of Se against miscarriage also observed seven- to nine-fold higher levels of Se in placental samples of miscarriage cases (mean 9.67–12 nmol/g) compared to the study from Poland that reported a detrimental effect (median 1.38 nmol/g) [25,26,29]. No direct comparison of placental Se levels can be made with the study from Turkey, as Se levels were reported in μg/L rather than ng/g or nmol/g.

**Table 2:** Summary of findings on the association between selenium and miscarriage risk in the included studies.

Study	Study Design (Sample Size)	Timing of Exposure Assessment	Main Findings	Major Limitations
Vinceti et al. 2000 [18]	Cohort study (*n* = 1128)	Throughout pregnancy	No significant association between exposure to the inorganic Se in tap water and risk of miscarriage.	Low statistical power (only 23 exposed pregnancies). Even in the exposed pregnancies, Se levels in tap water were below the WHO limit of 10 μg/L.
Jie O. et al. 2019 [19]	Case-control study (*n* = 195)	During each visit to the hospital (exact gestational stage not specified)	No significant association between Se levels in maternal blood and urine and risk of miscarriage.	Potential confounding by gravity and parity status.
Baser et al. 2020 [20]	Case-control study (*n* = 90)	At miscarriage or delivery (Cases: 12–20 weeks Controls: full-term)	Higher Se levels in placenta were associated with higher risk of miscarriage at 2nd trimester.	Reverse causality cannot be excluded. Potential confounding by gestational age. Low statistical power (30 miscarriage cases).
Abdulah et al. 2013 [21]	Case-control study (*n* = 71)	Between 8 and 20 weeks of gestation	Lower Se levels in maternal blood were associated with higher risk of miscarriage.	Low statistical power (25 miscarriage cases).
Zachara et al. 2001 [22]	Case-control study (*n* = 76)	3–23 weeks of pregnancy	No significant association between Se levels in maternal blood and plasma and risk of miscarriage.	Reverse causality cannot be excluded. Low statistical power (40 miscarriage cases).
Barrington et al. 1996 [23]	Case-control study (*n* = 80)	During 1st trimester checkup	Lower Se levels in maternal serum were associated with higher risk of miscarriage.	Reverse causality cannot be excluded. Low statistical power (40 miscarriage cases).
Koçak et al. 1999 [24]	Case-control study (*n* = 40)	During 1st trimester checkup	Lower Se levels in maternal serum were associated with higher risk of recurrent miscarriage at 1st trimester.	Low statistical power (20 miscarriage cases).
Ghneim et al. 2016 [25]	Case-control study (*n* = 50)	At miscarriage or delivery (Cases: 12.6 ± 3.4 weeks)	Lower Se levels in maternal blood, plasma, and placenta were associated with higher risk of recurrent miscarriage at 1st trimester.	Reverse causality cannot be excluded. Potential confounding by gestational age. Low statistical power (25 miscarriage cases).
Omeljaniuk et al. 2015 [26]	Case-control study (*n* = 118)	At miscarriage or delivery and 1st trimester. (Cases: 8.9 weeks)	Higher Se levels in maternal serum and placenta were associated with higher risk of miscarriage.	Reverse causality cannot be excluded. Potential confounding by gestational age and BMI.
Hu Y. et al. 2023 [27]	Case-control study (*n* = 200)	On the 14th day after embryo transfer	Lower Se levels in maternal serum were associated with higher risk of miscarriage after IVF.	-
Mishra et al. 2003 [28]	Case-control study (*n* = 97)	Not specified	No significant association between Se levels in maternal blood and plasma and risk of miscarriage.	Reverse causality cannot be excluded. Potential confounding by gestational age cannot be assessed. Low statistical power (52 miscarriage cases).
Al-Sheikh et al. 2019 [29]	Case-control study (*n* = 56)	At miscarriage or delivery (Cases: 12.6 ± 2.8 weeks)	Lower Se levels in maternal plasma and placenta were associated with higher risk of recurrent abortion.	Reverse causality cannot be excluded. Potential confounding by gestational age. Low statistical power (28 miscarriage cases).
Güvenç et al. 2002 [30]	Case-control study (*n* = 48)	At miscarriage or delivery	Lower Se levels in maternal serum and hair were associated with higher risk of miscarriage.	Reverse causality cannot be excluded. Potential confounding by gestational age. Low statistical power (16 miscarriage cases).
Desai et al. 2006 [31]	Case-control study (*n* = 60)	Cases: 12.8 weeks (at miscarriage) Controls: 13.2 weeks	Lower Se levels in maternal red cells were associated with higher risk of miscarriage. Higher Se levels in maternal plasma were associated with higher risk of miscarriage.	Reverse causality cannot be excluded. Low statistical power (30 miscarriage cases).

Twelve studies in total, involving 526 miscarriage cases and 509 controls, have examined whether the levels of Se in maternal whole blood, plasma, or serum differed between miscarriage cases and controls (Table 2). Of these, seven studies reported lower Se levels in miscarriage cases [21,23,24,25,27,29,30], two studies reported higher Se levels [26,31], while three studies found no difference [19,22,28]. Concerns regarding confounding by gestational age are present for three studies reporting a protective effect of Se and one reporting a detrimental effect [25,26,29,30]. Desai et al. reported higher plasma Se levels in miscarriage cases, indicative of a detrimental effect, and observed at the same time lower levels of Se in maternal red cells [31]. The study by Hu et al. has also explored whether a dose-response effect was present by fitting logistic regression models with Se levels divided into quartiles, and observed a protective effect of Se on miscarriage risk in a dose-response manner [27]. It should be noted that all studies had similar sample sizes (ranging from 40 to 200) and reported similar average Se levels, and thus, heterogeneity of results is unlikely to be explained by these factors.

There were also two studies that analyzed Se levels in other maternal samples (Table 2) [19,30]. Jie et al. found no difference in urine Se levels between 95 miscarriage cases and 100 controls [19]. Güvenç et al. observed lower Se levels in hair samples of 16 women that miscarried compared to 32 controls [30]. It should be noted that in the latter study, cases and controls differed in terms of gestational age; case samples were obtained shortly after miscarriage occurring prior to 20 gestational weeks, whereas control samples were obtained shortly after normal delivery [30]. In addition, hair samples from the proximal 1–2 cm were used, which reflect cumulative medium-term exposure, corresponding to first trimester or periconception period for miscarriage cases and third trimester for controls [30].

Of the reviewed studies, seven studies have additionally explored the GPx activity, as a functional indicator of Se status, in relation to miscarriage, among 283 miscarriage cases and 245 controls (Table 3). Five of these studies reported that GPx activity is reduced among miscarriage cases, indicating a protective effect of Se against miscarriage [22,25,28,29,31], one study reported increased GPx activity [26], while one study did not find any statistically significant difference [21]. The five studies reporting a protective effect of Se against miscarriage examined GPx activity in plasma or serum that reflects short-term Se status [22,25,28,29,31], while four studies additionally examined GPx activity in red cells or whole blood that reflect long-term Se status [22,25,28,31]. Lastly, two studies examined placental GPx activity and again reported a protective effect of Se against miscarriage [25,29]; however, both studies are prone to bias due to confounding by gestational age as control placental samples were obtained at term, and, therefore, conclusions regarding local protection against ROS toxicity in the feto-maternal system cannot be drawn.

With regard to overall confidence in the body of evidence presented above, it was downgraded to low mainly due to methodological limitations, inconsistency in results, and reduced coherence across studies. A major methodological concern in multiple studies was the confounding due to differences in gestational age between miscarriage cases and controls. Furthermore, all data arose from observational studies, while no randomized controlled trial was identified. Directional inconsistency was also observed especially in the studies of maternal blood Se levels that could not be explained by differences in average Se levels in the study population. Lastly, imprecise effect estimates were observed especially in studies with lower statistical power due to limited sample size and/or small number of miscarriage cases.

## 4. Discussion

In this systematic review of the literature, we qualitatively synthesized the evidence regarding the effect of Se exposure during pregnancy and maternal Se status on miscarriage risk. Despite apparent limitations in some of the reviewed studies, the available scientific evidence suggests that blood Se levels are probably lower in women who miscarry compared to women that have an uncomplicated pregnancy. It is, therefore, likely that Se exerts a protective effect against miscarriage. Nonetheless, causality has not been established, as all the available evidence arises from observational, mostly case-control, studies, while no randomized controlled trial exploring the effect of Se supplementation has been identified.

### 4.1. Miscarriage: Pathophysiological Mechanisms Relevant to Selenium

Miscarriage is defined as the spontaneous abortion occurring before 20 weeks of gestation [33]. Its etiopathogenesis is multifactorial, involving genetic and endocrine abnormalities, uterine structural pathology, infectious agents, autoimmune disorders, thrombophilia, as well as nutritional and environmental influences [33]. Nevertheless, in approximately 50% of recurrent miscarriage cases, the underlying cause remains unidentified [33]. In the context of this systematic review, particular emphasis is placed on factors associated with oxidative stress, ferroptosis, immunological dysregulation, and thyroid autoimmunity. Oxidative stress contributes to embryonic and pregnancy loss through impairment of placental function, induction of apoptosis, and activation of inflammatory pathways [34]. At the placental level, ROS induce endothelial dysfunction, reduce nitric oxide (NO) synthesis, and promote vasoconstriction, thereby compromising the placental supply of oxygen and nutrients [34]. Furthermore, ROS induce mitochondrial dysfunction, activate caspases, and deplete antioxidant defenses, culminating in ROS-driven apoptosis within placental tissues [34]. Additionally, ROS activate the NF-κB signaling cascade, leading to increased production of pro-inflammatory cytokines such as TNF-α, IL-6, and IL-1β, underscoring the interplay between oxidative stress and immunological factors [34]. Under physiological conditions, appropriate placental development and immune tolerance toward the semi-allogeneic embryo are essential for pregnancy maintenance. Perturbations in several immune components have been implicated in miscarriage, particularly in recurrent cases. Uterine natural killer (NK) cells, which normally facilitate trophoblast invasion and vascular remodeling, exhibit enhanced cytotoxicity or abnormal activation in recurrent miscarriage cases [35]. Regulatory T cells, crucial for establishing immune tolerance to the semi-allogeneic embryo, are reduced in number or functional capacity [35]. In addition, dysregulated antigen presentation by dendritic cells contributes to impaired maternal immune tolerance [35]. Finally, an excessively pro-inflammatory uterine environment, characterized by an increased M1/M2 macrophage ratio, decreased anti-inflammatory cytokines (e.g., IL-10, TGF-β), and elevated pro-inflammatory cytokines (e.g., TNF-α, IL-17), further compromises immune tolerance and placental development, ultimately heightening the risk of miscarriage [35]. Recently, the involvement of ferroptosis in miscarriage has gained increasing recognition; ferroptosis is a form of regulated cell death driven by iron-dependent lipid peroxidation, which may contribute to defective implantation through aberrant cell death within the decidua [36]. Complex interactions exist among ferroptosis, oxidative stress, and immune regulation [36]. Ferroptosis is promoted under conditions of glutathione depletion, which leads to inactivation of GPx4, reduced detoxification of phospholipid hydroperoxides, and subsequent accumulation of lipid-derived ROS [36]. Furthermore, pro-inflammatory cytokines can enhance ferroptotic processes, whereas anti-inflammatory cytokines exert a protective, inhibitory effect [36]. An additional link has been demonstrated between ferroptosis and macrophage polarization, as alterations in the M1/M2 macrophage ratio appear to modulate ferroptotic activity both intracellularly and through intercellular signaling [36]. Lastly, thyroid autoimmunity is recognized as a risk factor for recurrent pregnancy loss, even in the absence of overt or subclinical hypothyroidism [37]. This increased risk appears to persist irrespective of levothyroxine therapy and is evident in both first- and second-trimester miscarriages [37].

### 4.2. Selenium and Regulation of Oxidative Stress, Immune System, Ferroptosis, and Thyroid Hormones

**Figure 2:**
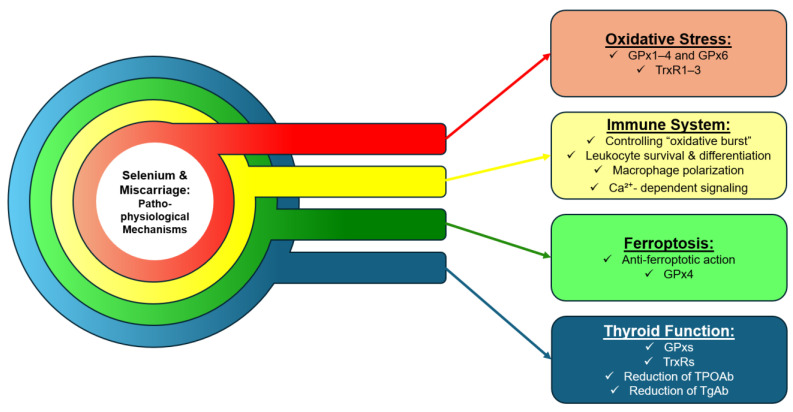
Pathophysiological mechanisms linking selenium to miscarriage. GPx: Glutathione peroxidase; TrxR: Thioredoxin Reductase; TPOAb: Thyroid Peroxidase Antibodies; TgAb: Thyroglobulin Antibodies.

Selenium plays a pivotal role in maintaining redox homeostasis through its incorporation into selenoproteins. The principal selenoproteins involved in antioxidant defense include the GPxs and thioredoxin reductases (TrxRs), encompassing GPx1–4 and 6, and TrxR1–3 (Figure 2). In contrast, GPx5 and GPx7–8 lack selenocysteine residues and instead contain cysteine in their catalytic sites, rendering their enzymatic activity independent of Se [38]. Antioxidant selenoproteins neutralize harmful free radicals via reduction reactions. Specifically, GPxs catalyze the conversion of reactive hydrogen peroxide (H_2_O_2_) into water and oxygen, GPx4 additionally reduces membrane lipid hydroperoxides, and TrxRs reduce protein disulfides [39]. Maintaining balanced levels of ROS and reactive nitrogen species (RNS) is critical for preventing oxidative damage in healthy tissues and supporting optimal immune responses [38]. With regard to animal data, Li et al. (2021) employed a porcine model to investigate the impact of organic selenium-enriched diets on antioxidant capacity [40]. In the experimental group, expression of GPxs and TrxRs in gastrointestinal tissues increased, while levels of malondialdehyde (MDA), a biomarker of lipid peroxidation, showed a downward trend [40]. Moreover, the selenium-supplemented group exhibited reduced concentrations of pro-inflammatory cytokines IL-6 and TNFα, alongside elevated IL-2 levels, indicating an anti-inflammatory response [40]. Collectively, these findings suggest that organic Se supplementation may exert beneficial antioxidant and immunomodulatory effects.

Selenium is integral to regulating immune responses through its involvement in redox balance and cellular signaling (Figure 2). Selenoproteins, including SelK and GPxs, are essential for controlling the “oxidative burst”, a process in which leukocytes rapidly produce ROS to eliminate pathogens, while selenium’s antioxidant properties prevent excessive ROS accumulation that could damage host tissues [41]. Selenium also influences leukocyte survival and differentiation, promoting the transition of macrophages from a pro-inflammatory (M1) to an anti-inflammatory (M2) phenotype, thereby supporting immune tolerance [42]. Additionally, Se mediates Ca^2+^-dependent signaling critical for macrophage phagocytic activity and can reduce leukocyte adhesion to endothelial cells, modulating inflammatory cell trafficking [41]. Collectively, these mechanisms contribute to balanced immune regulation and are particularly important for maintaining maternal-fetal tolerance, potentially reducing the risk of immunologically mediated pregnancy complications such as miscarriage. However, selenium’s effects are context-dependent. Ghaniem et al. reported that in Nile tilapia with Se deficiency, administration of 1 mg/kg inorganic Se increased biomarkers of innate immune function but also elevated mRNA expression of pro-inflammatory cytokines, including TNFα, TGFβ1, and IL1β, indicating that Se can also exert immunostimulatory effects [43]. No changes were observed in circulating leukocyte populations, such as monocytes, eosinophils, or basophils. These findings suggest that, although Se enhances antioxidant and anti-inflammatory capacity, it may initially trigger an inflammatory immune response, which could be harmful in certain contexts.

Selenium is also an important regulator of ferroptosis, a form of regulated cell death. The discovery that the glutathione (GSH)-dependent selenoenzyme GPx4 detoxifies membrane-bound phospholipid hydroperoxides underscores the central role of the GPx4–GSH–cysteine axis in preventing ferroptosis (Figure 2) [44]. Specifically, Se is notable for the activity of ebselen, a synthetic organoselenium compound, which can inhibit ferroptosis induced by erastin exposure or GPx4 deletion [45,46], highlighting selenium’s antioxidative and anti-ferroptotic properties. The decidua, a specialized endometrial tissue, plays a key role in embryo implantation and immune regulation, and dysregulation of ferroptosis in this tissue can impair implantation or immune function, potentially leading to early pregnancy loss [36]. Overall, Se, through its anti-ferroptotic action, may contribute to the protection of decidualization and the maintenance of pregnancy. 

Lastly, Se is involved in maintaining thyroid health (Figure 2). Through its involvement in antioxidant defense, immune regulation, and thyroid hormone metabolism, Se is an important regulator of thyroid homeostasis. As a key component of GPxs and TrxRs, Se protects thyroid tissue from oxidative stress, a major contributor to the pathophysiology of thyroid autoimmunity [47]. Multiple studies have shown that Se supplementation, particularly in individuals with Se deficiency, reduces thyroid autoantibody titers, notably TPOAb and TgAb, in patients with Hashimoto’s thyroiditis, other autoimmune thyroid diseases, or no prior thyroid pathology [48,49,50]. However, the therapeutic range of Se is narrow, and supplementation should be guided by baseline Se status and individual metabolic variation to avoid toxicity [51].

### 4.3. Supportive Data Emerging from Animal Model Studies

Several animal model studies have provided extensive evidence of selenium’s dual role as both an essential micronutrient and a potential teratogen, depending on its chemical form, dosage, and species sensitivity. Studies have shown that selenomethionine and selenite are generally more teratogenic than selenate, with notable interspecies variability in susceptibility to selenium-induced toxicity [52,53,54,55,56,57]. Studies in rodents have explored the potential teratogenicity of Se and its effect on absorption rates, by administering very high doses of selenite and/or selenate, by exploring its impact when co-exposure with methylmercury occurs, or by administering it through continuous infusion [53,54,58,59,60]. Notably, Hau et al. and Danielsson et al. observed a detrimental effect only in high doses [53,59] and Ferm et al. only when infused continuously but not when administered orally [54]. In addition, all the aforementioned studies administered inorganic Se, as selenite or selenate; it is, however, known that each of these forms exhibits distinct absorption capacities, metabolic routes, and toxicity characteristics [61]. In addition, dietary intake is the main contributor for Se intake in humans, and organic rather than inorganic Se forms are dominant in foods [61]. In contrast to rodent studies, studies in mammals, namely sheep, have shown that Se supplementation can lead to decreased abortion rates [62,63] and that lower plasma Se levels are observed in aborted cases [64], indicating a protective effect of Se against miscarriage. Nonetheless, evidence of embryotoxicity at subtoxic doses has also been reported in sheep treated with selenate and selenite [65]. In total, these findings underline the limitations of animal models in studying Se impact on miscarriage risk and translating these findings for humans, as well as the importance of maintaining Se within the narrow normal range to achieve reproductive success.

### 4.4. Dynamic Changes of Selenium Concentration During Gestation

Selenium concentrations in biological samples undergo dynamic changes throughout pregnancy, a factor that is essential to consider when interpreting studies assessing the association between Se levels and miscarriage risk. Selenium is primarily excreted via urinary and fecal routes, with minor elimination occurring through breath, saliva, and hair [66]. Regarding urinary excretion, Se is predominantly eliminated through the proximal renal tubules, where methylated Se metabolites are actively secreted into the tubular lumen following initial glomerular filtration [67]. During pregnancy, the glomerular filtration rate (GFR) typically increases by approximately 40–50% above pre-pregnancy values during the second trimester, while proximal tubular reabsorption decreases [68,69]. Concurrently, as embryonic circulation and oxygen-dependent metabolism are established early in gestation, the developing embryo relies increasingly on maternal antioxidant defenses transferred via the placenta [70]. These physiological adaptations, together with maternal plasma volume expansion and subsequent hemodilution [71], result in a progressive decline in maternal blood Se concentrations to levels well below those observed before conception [72,73,74]. Therefore, when investigating Se status in relation to pregnancy outcomes, it is imperative that comparisons are made using samples collected at comparable gestational ages. Failure to account for gestational age-related variations in Se levels may lead to spurious differences between miscarriage cases and controls that merely reflect physiological changes inherent to pregnancy.

### 4.5. Strengths and Limitations of the Study

Our systematic review has provided a qualitative synthesis of scientific literature on the role of Se status during pregnancy in miscarriage, raising awareness of potentially modifiable risk factors for miscarriage. Taking into account that miscarriage affects 10–20% of all recognized pregnancies, an effect of Se status on miscarriage risk, even small, may have a considerable public health impact [8]. Our systematic review is characterized by a rigorous and broad search strategy that aimed to identify studies exploring the relationship between Se and several pregnancy and neonatal outcomes. We further presented evidence in support of the biological plausibility and the biochemical rationale linking Se to miscarriage, such as its role in antioxidant defense, ferroptosis, immune system regulation, and thyroid function. Integrating mechanistic and clinical evidence strengthens causal inference by underscoring the plausibility of the observational associations [75]. Our systematic review has also highlighted research gaps that need to be addressed for the development of high-grade evidence-based guidance.

Our systematic review has also several limitations, owing to its design but also to inherent limitations of the reviewed studies. First, this systematic review was not registered; nonetheless a study protocol was prepared for the SEduP project and will be made available upon request. Our search strategy did not cover every available database, but it was limited to PubMed and Embase; databases, such as Web of Science, that include more journals were not searched. Nevertheless, in the context of clinical research, medicine, health and life sciences, PubMed and Embase are expected to suffice [76]. In addition, our search strategy was not optimized to detect papers regarding GPx activity; thus, we cannot exclude the possibility that further studies exist examining only GPx activity and not Se supplementation or Se status during pregnancy in relation to miscarriage risk. Our conclusions are limited by the fact that all evidence arises from observational studies and there has been no randomized controlled trial on the topic, while power remains low as only 14 studies are included in the systematic review. Furthermore, in the real world pregnant women are not exposed to single substances, but rather to a mixture of chemicals and lifestyle factors that interact and may lead to effect modifications. With regard to Se, it is well known that deficiency of vitamin E and Se may have a synergistic effect [75]. In addition, Se can provide protection against several heavy metals (e.g., cadmium, silver, and mercury) in marine food, but on the other hand co-exposure can reduce its bioavailability [75]. Lifestyle factors also interact with Se; active and passive smoking increases oxidative stress and potentially the need for Se, while rigorous exercising may also lead to increased needs [75]. Despite the aforementioned, only one of the reviewed studies explored the potential role of co-exposure to heavy metals [20], and one the role of smoking [21]. Similarly, the impact of potential confounders has not been accounted for in many of the reviewed studies. In particular, no adjustment was applied in seven out of 14 studies, maternal age was accounted for only in six out of 14 studies, BMI in one, and smoking in one. Notably, confounding by gestational age was present in five out of the 14 reviewed studies, as cases and controls differed significantly in gestational age at sampling. Lastly, there is a marked lack of data for placental Se and GPx activity that is not affected by confounding due to gestational age; appropriate controls in case-control study design examining miscarriage are not women at term after delivery, but women that undergo elective abortion at similar gestational age.

### 4.6. Implications for Future Research

Taking into consideration the identified research gaps and the limitations of the studies conducted to date, there is need for both interventional and observational studies to be conducted to elucidate the role of Se in miscarriage. Randomized controlled trials on Se supplementation in the periconceptional period and in early pregnancy are required to strengthen the causality of the protective effect of Se against miscarriage reported by observational studies. Owing to the randomization, in such studies confounding is well controlled for. In addition, consistency of results across populations with different dietary patterns and environmental exposures would provide evidence in favor of a global effect of Se on miscarriage risk, even when the co-exposures to other environmental, nutritional, and lifestyle factors differ significantly, while populations with increased needs for Se can be identified by comparing effect sizes. With regard to observational studies, the association between maternal and placental Se levels in pregnancies that end with miscarriage, but also in uncomplicated pregnancies at term and elective abortions, need to be clarified in order to understand the local protection against ROS toxicity in the feto-maternal system that is offered by Se. Furthermore, the role of placental Se levels needs to be explored while using appropriate controls, namely elective abortions and not post-delivery placental samples that give rise to biased results by gestational age. Lastly, the possibility of synergistic effects of Se with other environmental, nutritional, and lifestyle exposures is to be explored. That said, well designed case-control studies to explore all the aforementioned aspects are difficult to implement, as pregnant women are usually visiting a health care professional during the first trimester rather than before conception, leading to recruitment challenges. Nonetheless, there are populations for which recruitment before conception and control for potential confounders is easier, as in the case of couples receiving assisted reproduction services. Studies among infertile couples would allow us to study maternal but not placental Se levels and GPx activity in relation to miscarriage, while also exploring if paternal Se status has any impact.

## 5. Conclusions

Adequate maternal Se status during early gestation likely ameliorates miscarriage risk, by providing protection against oxidative stress and ferroptosis, by contributing to immune system regulation, and by modulating thyroid autoimmunity and function. However, causality is not established, owing to the lack of randomized interventional data.

## Figures and Tables

**Table 1:** The search strategy in PubMed.

(“pregnancy”[MeSH Terms] OR “pregnan*”[Title/Abstract] OR “gestation”[Title/Abstract] OR “prenatal”[Title/Abstract] OR “intrauterine”[Title/Abstract] OR “in utero”[Title/Abstract] OR “perinatal”[Title/Abstract] OR “postnatal”[Title/Abstract])
AND
(“selenium” [MeSH Terms] OR “selenium” [tiab])
NOT
(“animals”[MeSH Terms] NOT “humans”[MeSH Terms])

* denotes truncation and retrieves all terms that begin with the specified word root.

**Table 3:** Summary of findings on the association between GPx activity and miscarriage risk in the included studies.

Study	Study Design (Sample Size)	Timing of Exposure Assessment	Main Findings	Major Limitations
Zachara et al. 2001 [22]	Case-control study (*n* = 76)	3–23 weeks of pregnancy	Lower GPx activity in maternal red cells and plasma was associated with higher risk of miscarriage.	Reverse causality cannot be excluded. Low statistical power (40 miscarriage cases).
Omeljaniuk et al. 2015 [26]	Case-control study (*n* = 118)	At miscarriage or delivery and 1st trimester. (Cases: 8.9 weeks)	Higher GPx activity in maternal serum was associated with higher risk of miscarriage.	Reverse causality cannot be excluded. Potential confounding by gestational age and BMI.
Mishra et al. 2003 [28]	Case-control study (*n* = 97)	Not specified	Lower GPx activity in maternal red cells and plasma was associated with higher risk of miscarriage.	Reverse causality cannot be excluded. Potential confounding by gestational age cannot be assessed. Low statistical power (52 miscarriage cases).
Al-Sheikh et al. 2019 [29]	Case-control study (*n* = 56)	At miscarriage or delivery (Cases: 12.6 ± 2.8 weeks)	Lower GPx activity in maternal plasma and placenta was associated with higher risk of recurrent miscarriage.	Reverse causality cannot be excluded. Potential confounding by gestational age. Low statistical power (28 miscarriage cases).
Desai et al. 2006 [31]	Case-control study (*n* = 60)	Cases: 12.8 weeks (at miscarriage) Controls: 13.2 weeks	Lower GPx activity in maternal red cells and plasma was associated with higher risk of miscarriage.	Reverse causality cannot be excluded. Low statistical power (30 miscarriage cases).
Abdulah et al. 2013 [21]	Case-control study (*n* = 71)	Between 8 and 20 weeks of gestation	No significant association between GPx activity in maternal serum and the risk of miscarriage.	Low statistical power (25 miscarriage cases).
Ghneim et al. 2016 [25]	Case-control study (*n* = 50)	At miscarriage or delivery (Cases: 12.6 ± 3.4 weeks)	Lower GPx activity in maternal plasma, blood, and placenta was associated with higher risk of recurrent miscarriage.	Reverse causality cannot be excluded. Potential confounding by gestational age. Low statistical power (25 miscarriage cases).

## Data Availability

No new data were created or analyzed in this study. Data sharing is not applicable to this article.

## Supplementary Materials

The following supporting information can be downloaded at: https://www.mdpi.com/article/10.3390/ijms27020968/s1.
